# Thermodynamic Operations and Entropy Considerations for a Ring-of-Charge Oscillator System

**DOI:** 10.3390/e28010019

**Published:** 2025-12-24

**Authors:** Daniel C. Cole

**Affiliations:** Department of Mechanical Engineering, Boston University, Boston, MA 02215, USA; dccole@bu.edu

**Keywords:** classical physics, harmonic oscillator, thermodynamics, electromagnetic radiation, electrodynamics, stochastic electrodynamics, zero point

## Abstract

A ring of classical charge with a charged point particle oscillating within is first analyzed. The charged particle interacts with classical electromagnetic thermal radiation, which causes the particle to fluctuate, while the ring of charge imparts a resonant frequency to the particle’s motion. Oscillations in one direction within the plane of the ring are analyzed. The radius of the ring is slowly altered. The accompanying change in the particle’s average internal energy and the average work done in changing the radius are calculated. This leads to a derivation of the classical electromagnetic zero-point radiation spectrum. Next, the second law of thermodynamics is applied to the entropy to enable a more general derivation of the Wien displacement law. With this derivation, zero-point radiation can be included in the Wien displacement law. Finally the definition of the thermodynamic temperature is emphasized, and methods for performing the needed calculations for the temperature ratio are discussed.

## 1. Introduction

This article introduces a simple classical electrodynamic oscillator system that interacts with classical electromagnetic thermal radiation. This system is then used to prove several results of interest regarding electromagnetic thermal radiation. In particular, a thermodynamic analysis will enable us to derive classical electromagnetic radiation at absolute zero temperature. This nonzero radiation at T=0 is called classical electromagnetic zero-point (ZP) radiation.

The system analyzed here is a ring of charge, within which a charged point particle fluctuates due to its interactions with classical electromagnetic radiation. Changing the radius of the ring of charge alters the resonant frequency imposed on the fluctuating particle’s motion.

What is important about being able to derive ZP radiation is that it is central to the classical theory of nature called stochastic electrodynamics (SED) [1,2,3]. SED is indeed a classical theory in that it is based on two things: (1) Maxwell’s microscopic classical equations of electrodynamics and (2) the classical and relativistic version of Newton’s second law of motion for point charges or, in other words, the Lorentz–Dirac equation [4,5].

SED has deduced a surprising number of phenomena normally thought explainable only via quantum mechanics (QM) and quantum electrodynamics (QED). Examples of such phenomena are as follows: Casimir forces [1,6], van der Waals forces [7], a close agreement between classical and quantum mechanical SHOs [8], diamagnetism [9], thermodynamics of radiation in a cavity [10], and thermal effects of acceleration [11,12,13].

Many of these calculations in QM and QED are typically carried out in the ground state, which, in SED, means at T=0, ZP radiation comes into play here, since at T=0 is where ZP radiation is used for calculating Casimir forces, van der Waals forces, and even the thermal effects of acceleration (i.e., constant acceleration through ZP radiation). SED also calculates phenomena for T>0 situations that agree with QM, but it uses the natural continuum to classical electromagnetic zero-point plus Planckian (ZPP) radiation, such as for calculating van der Waals forces in Ref. [7]. Other examples in SED that contain calculations in both ZP and ZPP (T>0) situations are provided in [6,8,10].

Thus, being able to derive the spectrum for ZP radiation is important for the basis of SED. Our derivation uses a relatively simple electrodynamic system. In principle, any other physical system should work just as well, as all should follow the basic idea that no heat flows at T=0 for isothermal and reversible physical operations. However, for most systems, such an analysis is difficult to carry out. An example of a much more complicated system is in Ref. [14]. Our proof here is much simpler and transparent as to the basic physics, as it deals directly with changing the resonant frequency of the system.

We will consider a ring of charge that provides a simple harmonic oscillator (SHO) potential to the fluctuating point charge. This SHO potential creates a resonant frequency component to the particle’s motion. To simplify the situation, the particle will be confined to oscillate in the plane of the ring, and motion will be restricted to one linear direction through the center of the ring. We will analyze the situation in which the ring’s radius is increased or decreased and deduce how this affects the oscillating charged particle as it interacts with the thermal radiation.

Two key quantities calculated in this study are the internal energy of the oscillator system and the work done to change the radius of the ring of charge with the point charge being within the ring. These calculations will be carried out in Section 2.2 and Section 2.3.

Then, we proceed with calculating the average, or the expectation value, of these two quantities. To this end, some background on the stochastic process involved is required. This topic is covered in Section 3.

In Section 4, we will impose that the heat *Q* is zero, which flows either in or out of a large volume *V* that encloses the ring oscillator system. This zero value is made to hold while the oscillator ring is slowly changed to larger or smaller sizes. This is where the derivation of ZP radiation takes place.

In Section 5, we will apply the second law of thermodynamics for entropy and derive, for our oscillator system, a result that the ring-of-charge oscillator system must obey for all temperature and radius values. This result will be used in Section 6 to obtain a generalized Wien displacement law. This more general derivation allows for ZP radiation, while the conventional one by Wien and as published by Planck [15] does not.

In Section 7, some comments will be made about applying the previous material to adiabatic processes and achieving the right heat flow in isothermal processes, bounded by adiabatic ends, to obtain a ratio of heat flow that equals the corresponding temperature ratio.

Section 8 ends with some concluding remarks.

## 2. Internal Energy and “Work Done” While Changing the Ring-of-Charge Radius

### 2.1. Overview

As a reminder, the reason for pursuing this ring of charge with a point charge that oscillates/fluctuates inside it is that this simple but unusual oscillator system, interacting with thermal radiation, can be used to derive some interesting quantities.

The structure of this oscillator system is simply a circular ring of charge with a radius *a*, where a classical charged point particle with charge *q* and mass *m* is located near the ring’s center. The charge density of the ring will be the constant charge per unit length, λ, around the ring such that 2πaλ=q. Both the ring and point charge have the same sign of charge and, in this case, the same net charge of *q*. As the ring’s radius changes, λ also clearly changes, but in a manner such that 2πaλ always stays equal to the fixed value of *q*.

As the point charge approaches a side of the ring, it is pushed back to the center of the ring; the closer the particle to the ring, the stronger this push. For small displacements, the potential energy of the charge *q* inside the ring is that of an SHO potential, as will be shown.

As noted in Section 1, we will simplify this problem by assuming that *q* only moves, or fluctuates, along the *x*-axis in the plane of the ring of charge. This can be pictured by imagining a very thin rod along *x* that *q* slides along with no friction. However, it should also be noted that it would not be very difficult to change this system into a two-dimensional oscillating particle, since the ring of charge creates a two-dimensional SHO potential.

In the case of internal energy, this will consist of three things: (1) the potential energy of the fluctuating point charge in relation to the ring of charge, (2) the kinetic energy of the oscillating charge, and (3) the electromagnetic radiation energy within a large volume *V* that encases the oscillating system. Although the third item (radiation energy) could be important under certain circumstances, it is not important here, as the process of changing the ring’s size occurs under isothermal conditions. Hence, the radiation energy in *V* will change very little during the displacement operation (i.e., changing the size of the ring). However, what will change is that radiation can flow in or out of *V* due to the displacement operation, depending on whether the ring is made larger or smaller.

Besides internal energy (specifically, the change in internal energy $\delta U_{\mathrm{int}}$), the work done to make the ring either larger or smaller will be calculated. These two quantities, internal energy $\delta U_{\mathrm{int}}$ and “work done” *W*, are of interest, as they occur in the first law of thermodynamics along with *Q*, which is the heat flow in or out of *V*:

(1) $$\delta U_{\mathrm{int}}=Q+W\;\;.$$

All these quantities are averages or expectation values. The sign convention we will follow is that *Q* is positive if heat flows **into** *V*, thereby making $\delta U_{\mathrm{int}}$ larger (and negative otherwise); similarly, *W* is positive if work is done **on** the oscillator system, thereby also making $\delta U_{\mathrm{int}}$ larger (and negative otherwise) [16].

The work done, W, is a harder quantity to calculate than the change in internal energy $\delta U_{\mathrm{int}}$. As the point charge comes closer to one side of the charged ring, the ring tends to deform as *q* acts to push the ring. We will imagine external forces that are applied to keep the ring in a circle of radius *a*, even when the charge *q* approaches a wall. In addition, the external forces will be the entities that make the ring’s circle grow smaller or larger. The operation is a little subtle, as at each angle of the circle, the applied external force will, in general, differ based on the relative position of the fluctuating charge *q* near the side of the ring. The work done in changing the radius *a* will equal the radial component of the external force on each charge increment in the ring, times δa, all summed up. It is important to note that our calculations will only entail the work on the ring due to the presence of the *q* charge, as opposed to the ring acting on itself.

In the two subsections that follow for internal energy and “work done” (while changing *a*), we will be dealing with the expressions for these quantities prior to taking the average or expectation values. The latter will be carried out in Section 3.

### 2.2. Internal Energy

The easy part of calculating the internal energy is the (nonrelativistic) kinetic energy, as it is only

(2) $$U_{\mathrm{KE}}=\frac{m{\dot{x}}^{2}}{2}\;\;,$$

where $\dot{x}=\frac{dx}{dt}$.

**Figure 1:**
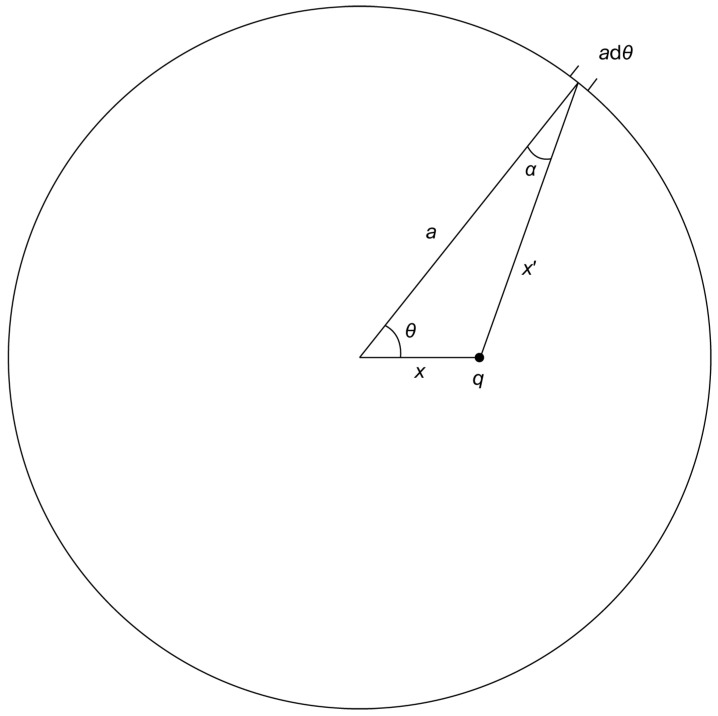
A diagram for computing (1) the potential energy and (2) the “work done” in changing *a*, the radius of the ring. θ is the angle from the horizontal axis and center of the ring to the segment adθ. $x^{\prime }$ is the distance from *q* to $a\left(d\theta \right)$. α is the angle between *a* and $x^{\prime }$; it will be used in the next subsection for computing the work done in changing *a*.

As for potential energy, Figure 1 shows the layout of how to carry out this calculation. The radius is, of course, *a*; *x* is the distance that the point charge *q* is from the center of the ring; $x^{\prime }$ is the distance from *q* to the indicated small increment $a\left(d\theta \right)$ on the circumference.

The electrostatic potential energy between *q* and the indicated small section of the circle is equal to

(3) $$U_{\mathrm{PE},d\theta }=\frac{q\left(\lambda ad\theta \right)}{x^{\prime }}\;\;.$$

where $x^{\prime }$ can be expressed in terms of *a*, *x*, and θ using the law of cosines:

(4) $${\left(x^{\prime }\right)}^{2}=a^{2}+x^{2}-2ax\cos\theta \;\;.$$

The total potential energy between *q* and the ring of charge then becomes

(5) $$U_{\mathrm{PE}}=\int_{0}^{2\pi }\frac{q\left(\lambda ad\theta \right)}{{\left(a^{2}+x^{2}-2ax\cos\theta \right)}^{1/2}}=q\lambda \int_{0}^{2\pi }\frac{d\theta }{{\left(1+f^{2}-2f\cos\theta \right)}^{1/2}}\;\;,$$

where $f=\frac{x}{a}$. Looking at the situation of f≪1 and expanding the integrand in terms of *f*, we obtain

(6) $$\begin{array}{cc} \; & {\left(1+f^{2}-2f\cos\theta \right)}^{-1/2}\\ \; & =1+f\cos\theta +f^{2}\left(\frac{3}{2}\cos^{2}\theta -\frac{1}{2}\right)-f^{3}\left[\frac{2}{3}\cos\theta -\frac{5}{3}\cos\theta \left(\frac{3}{2}\cos^{2}\theta -\frac{1}{2}\right)\right]+O\left(f^{4}\right)\;\;. \end{array}$$

Hence, up until the third order in *f*,

(7) $$\begin{array}{cc} U_{\mathrm{PE}} & \approx q\lambda \int_{0}^{2\pi }d\theta \left\{1+f\cos\theta +f^{2}\left(\frac{3}{2}\cos^{2}\theta -\frac{1}{2}\right)-f^{3}\left[\frac{2}{3}\cos\theta -\frac{5}{3}\cos\theta \left(\frac{3}{2}\cos^{2}\theta -\frac{1}{2}\right)\right]\right\}\\ \; & =q\lambda \left[2\pi +0+f^{2}\left(\frac{3}{2}\pi -\pi \right)+0\right]\\ \; & =\frac{q\left(\lambda 2\pi a\right)}{a}\left[1+f^{2}\frac{\pi }{2}\frac{1}{2\pi }\right]=\frac{q^{2}}{a}+\frac{q^{2}}{4a^{3}}x^{2}\;\;. \end{array}$$

The last term of $\frac{q^{2}}{4a^{3}}x^{2}$ signifies an SHO potential that we can equate to $\frac{m\omega_{0}^{2}}{2}x^{2}$, with the identification that

(8) $$\omega_{0}^{2}=\frac{q^{2}}{2a^{3}m}\;\;.$$

Hence,

(9) $$U_{\mathrm{PE}}=\frac{q^{2}}{a}+\frac{m\omega_{0}^{2}}{2}x^{2}\;\;.$$

The first term of $\frac{q^{2}}{a}$ in Equation (9) is related to the potential energy needed to place the *q* charge at the center of the ring of charge, or when x=0. This term will drop out when we complete our calculations for the “work done” material.

### 2.3. Work Done to Change the Radius of the Ring of Charge

Here, we will be concerned with the work done by external forces to keep the ring of charge in a circle and to expand or contract the ring by changing *a*, all while *q* is at some position *x* from the center of the ring.

We can make use of Figure 1 again, this time bringing in cosα, where α is the angle between (1) the direction from *q* to the infinitesimal charge λadθ and (2) the radial direction along *a*. The component of cosα projects the direction of *q* to λadθ onto the radial direction. This is important because we need the component of the external forces along the direction that the ring moves when expanded or contracted, which is the radial direction. More specifically, the incremental work done in moving λadθ is F·ds, where F is the external force, equal and opposite to the electrostatic force between *q* and λadθ (points along $x^{\prime }$ in the diagram), and ds is the infinitesimal displacement of λadθ along the radius. The angle in the dot product of F·ds is α, which is the angle between *a* and $x^{\prime }$ in Figure 1.

The work to make the ring of charge larger $\left(\delta a>0\right)$ or smaller $\left(\delta a<0\right)$ is done by external forces that are equal and opposite [hence the minus sign in Equation (10)] to the internal electrostatic forces of the system, thereby keeping the system together and/or being ever so slightly larger or smaller to slowly change the ring’s radius. The “work done” is expressed in Equation (10), where ${\left(x^{\prime }\right)}^{2}$ is given in Equation (4), and $\cos\left(\alpha \right)$, by the law of cosines, is given in Equation (11):

(10) $$W=-\delta a\int_{\theta =0}^{\theta =2\pi }\frac{q\left(\lambda ad\theta \right)}{{\left(x^{\prime }\right)}^{2}}\cos\left(\alpha \right)\;\;,$$

(11) $$\cos\alpha =\frac{a^{2}+{\left(x^{\prime }\right)}^{2}-x^{2}}{2ax^{\prime }}\;\;.$$

Substituting Equation (4) into Equation (11) yields

(12) $$\begin{array}{cc} \cos\alpha & =\frac{a^{2}+\left(a^{2}+x^{2}-2ax\cos\theta \right)-x^{2}}{2a{\left(a^{2}+x^{2}-2ax\cos\theta \right)}^{1/2}}\\ \; & =\frac{\left(a-x\cos\theta \right)}{{\left(a^{2}+x^{2}-2ax\cos\theta \right)}^{1/2}}\;\;. \end{array}$$

Equation (10) becomes

(13) $$\begin{array}{cc} W & =-\left(\delta a\right)q\lambda a\int_{0}^{2\pi }d\theta \frac{\left(a-x\cos\theta \right)}{{\left(a^{2}+x^{2}-2ax\cos\theta \right)}^{3/2}}\\ \; & =-\left(\delta a\right)\frac{q\lambda }{a}\int_{0}^{2\pi }d\theta \frac{\left(1-f\cos\theta \right)}{{\left(1+f^{2}-2f\cos\theta \right)}^{3/2}}\;\;. \end{array}$$

Expanding the integrand in Equation (13) in terms of *f*, we obtain

(14) $$\begin{array}{cc} \; & \frac{\left(1-f\cos\theta \right)}{{\left(1+f^{2}-2f\cos\theta \right)}^{3/2}}\\ \; & =1+2f\cos\theta +f^{2}\left(\frac{9}{2}\cos^{2}\theta -\frac{3}{2}\right)-f^{3}\left[4\cos\theta -\frac{4}{3}\cos\theta \left(\frac{15}{2}\cos^{2}\theta -\frac{3}{2}\right)\right]+O\left(f^{4}\right)\;\;. \end{array}$$

*W* then becomes, through the third order in *f*,

(15) $$\begin{array}{cc} W & \approx -\left(\delta a\right)\frac{q\lambda }{a}\int_{0}^{2\pi }d\theta \left\{1+2f\cos\theta +f^{2}\left(\frac{9}{2}\cos^{2}\theta -\frac{3}{2}\right)-f^{3}\left[4\cos\theta -\frac{4}{3}\cos\theta \left(\frac{15}{2}\cos^{2}\theta -\frac{3}{2}\right)\right]\right\}\\ \; & =-\left(\delta a\right)\frac{q\lambda }{a}\left[2\pi +0+f^{2}\left(\frac{9}{2}\pi -\frac{3}{2}2\pi \right)+0\right]=-\left(\delta a\right)\frac{q\left(2\pi \lambda a\right)}{a^{2}}\left[1+f^{2}\frac{3}{4}\right]\\ \; & =-\left(\delta a\right)\frac{q^{2}}{a^{2}}\left[1+\frac{3}{4}\frac{x^{2}}{a^{2}}\right]\;\;. \end{array}$$

Note that the ring is repelled outward by the +q point charge. When the ring is expanded, δa is positive, while the external force is inward to hold the ring together due to the *q* charge. The external force and δa are, therefore, in opposite directions, so the external force does negative work—hence the minus sign in Equation (15). When the ring is contracted, δa<0, so it is parallel to the external force; thus, W is positive in Equation (15).

The term $-\delta a\frac{q^{2}}{a^{2}}$ in Equation (15), which is independent of *x*, represents the work done by external forces due to the presence of *q* in changing the radius of the ring by δa, when the charge *q* is at the center of the ring.

Note that if Equation (7) for $U_{\mathrm{PE}}$ is differentiated with respect to *a*, one obtains Equation (15). In the next section, Section 3, we will be averaging over the $x^{2}$ term; then, in Section 4, the averaged quantity $\langle U_{\mathrm{PE}}\rangle$ will be differentiated with respect to *a*. This differentiation of the ensemble average will make a difference in the outcome of this relationship between the potential energy and the results of the work done.

## 3. Stochastic Process and Ensemble Averages

The interaction of the charge *q* with classical electromagnetic thermal radiation imparts a stochastic fluctuating motion to *q*, which is modified by the frequency resonance of the SHO potential. The values of *x* and $\dot{x}$ in the previous subsections, for $U_{\mathrm{KE}}$, $U_{\mathrm{PE}}$, and W, will fluctuate in time. To carry out the thermodynamic analysis in this article, we will need the average, or expectation value, of $U_{\mathrm{KE}}$, $U_{\mathrm{PE}}$, and W.

The mathematical means to carry out the needed stochastic analysis has been published and is available in Ref. [17]. Consequently, we will only summarize some of the material contained in this reference.

The electromagnetic radiation fields for thermal radiation will be represented as an infinite sum of plane waves, with periodic boundary conditions imposed. Fourier series will be used to describe the fields. The following sum of plane waves will be used for the electric $E\left(x,\;t\right)$ and magnetic $B\left(x,\;t\right)$ radiation fields in this large rectangular parallelepiped volume, within which the previously mentioned volume *V* is contained:

(16) $$E_{\mathrm{rad}}\left(x,\;t\right)=\frac{1}{{\left(L_{x}L_{y}L_{z}\right)}^{1/2}}\sum_{n_{x},n_{y},n_{z}=-\infty }^{\infty }\sum_{\lambda =1,2}{\hat{\varepsilon }}_{k_{n},\lambda }\left[\begin{array}{c} A_{k_{n},\lambda }\cos\left(k_{n}\cdot x-\omega_{n}t\right)\\ +B_{k_{n},\lambda }\sin\left(k_{n}\cdot x-\omega_{n}t\right) \end{array}\right]\;\;,$$

(17) $$B_{\mathrm{rad}}\left(x,\;t\right)=\frac{1}{{\left(L_{x}L_{y}L_{z}\right)}^{1/2}}\sum_{n_{x},n_{y},n_{z}=-\infty }^{\infty }\sum_{\lambda =1,2}\left({\hat{k}}_{n}{\times \hat{\varepsilon }}_{k_{n},\lambda }\right)\left[\begin{array}{c} A_{k_{n},\lambda }\cos\left(k_{n}\cdot x-\omega_{n}t\right)\\ +B_{k_{n},\lambda }\sin\left(k_{n}\cdot x-\omega_{n}t\right) \end{array}\right]\;\;,$$

where

(18) $$k_{n}=\frac{2\pi n_{x}}{L_{x}}\hat{x}+\frac{2\pi n_{y}}{L_{y}}\hat{y}+\frac{2\pi n_{z}}{L_{z}}\hat{z}\;\;,$$

$n_{x}$, $n_{y}$, and $n_{z}$ are integers. To satisfy all four of Maxwell’s classical electrodynamic equations for fields in free space, $\omega_{n}=c\left|k_{n}\right|$, $k_{n}\cdot {\hat{\varepsilon }}_{k_{n},\lambda }=k_{n}\cdot {\hat{\varepsilon }}_{k_{n},\lambda^{\prime }}=0$, and ${\hat{\varepsilon }}_{k_{n},\lambda }\cdot {\hat{\varepsilon }}_{k_{n},\lambda^{\prime }}=0$ for $\lambda \neq \lambda^{\prime }$, where λ and $\lambda^{\prime }$ indicate the linear polarization direction. Here, λ and $\lambda^{\prime }$ are indices that take on only two values; thus, for example, each might be represented by the values of 1 or 2. Moreover, ${\hat{k}}_{n}=k_{n}/\left|k_{n}\right|$; similarly, all other vectors with a “carrot” are meant to be unit vectors, and all quantities in bold are vectors.

The equation of motion for *q*, with mass *m*, is

(19) $$m\ddot{x}\left(t\right)=-m\omega_{0}^{2}x\left(t\right)+m\Gamma \dddot{x}\left(t\right)+qE_{\mathrm{rad},x}\left(x=0,\;t\right)\;\;,$$

where $\Gamma =\frac{2}{3}\frac{q^{2}}{mc^{3}}$, $m\Gamma \dddot{x}$ is the nonrelativistic expression for the radiation reaction of a charged point particle of mass *m* and charge *q*, and *c* is the speed of light. Equation (19) is a damped, driven simple harmonic oscillator (SHO) equation. The resonant frequency of the fluctuating charge +q is specified here as $\omega_{0}$ in Equation (8) from the SHO binding potential in Equation (7), $-m\omega_{0}^{2}x=-\frac{d}{dx}U_{\mathrm{PE}}$. The dipole approximation of $E_{\mathrm{rad},x}\left(x=0,\;t\right)$ is made in Equation (19) when evaluating the electric field component of the Lorentz force, and the magnetic field component of the Lorentz force is assumed to be much smaller in magnitude than $qE_{\mathrm{rad},x}\left(x=0,\;t\right)$, so it is ignored here.

The Fourier coefficients are probabilistically different from one ensemble member to another. More specifically, each set of fields in Equations (16) and (17), for fixed values of the Fourier coefficients, can be viewed as being in a large ensemble of such fields, and each ensemble member of the fields has a charge obeying Equation (19) within their space. Changing the Fourier coefficients results in choosing a different ensemble member. A probability distribution exists for the values of the Fourier coefficients. A natural assumption is made in that the $A_{1},\;\cdots ,\;A_{N},\;B_{1},\;\cdots ,\;B_{N}$ Fourier coefficients are independent random variables with a Gaussian probability density distribution given by the following (see p. 126 in Ref. [1]):

(20) $$P_{F}\left(A_{p}\right)=\frac{1}{\sqrt{2\pi {\left(\sigma_{p}\right)}^{2}}}\exp\left(-\frac{A_{p}^{2}}{2{\left(\sigma_{p}\right)}^{2}}\right)\;\;.$$

Here, the subscript *p* provides the index label of these variables, $A_{1},\;\cdots ,\;A_{N}$, which, in turn, is shorthand for the labels $k_{n},\;\lambda$. By symmetry, the same distribution in Equation (20) also holds for the Fourier coefficient $B_{p}$. The label “F” is added to specify that this probability density function $P_{F}$ refers to the Fourier coefficients. Moreover, $\sigma_{p}$ depends on the frequency $\omega_{n}$, as in Equation (18), with $\omega_{n}=c\left|k_{n}\right|$, in addition to the temperature *T*, with T≥0. Thus, we are dealing with $\sigma_{p}^{2}\to {\left[\sigma \left(\omega_{n},T\right)\right]}^{2}$, but we will assume that ${\left[\sigma \left(\omega_{n},T\right)\right]}^{2}$ is independent of the direction of $k_{n}$ and only dependent on its magnitude.

Reference [17] carries out most of the ensemble averaging aspects that we need here. More specifically, in the expression for $U_{\mathrm{PE}}$ in Equation (9) the quantity $x^{2}$ appears. This needs to be averaged over to obtain $\langle x^{2}\rangle$ and thereby also obtain $U_{\mathrm{PE}}=\langle U_{\mathrm{PE}}\rangle ,$ where the angled brackets means to take the ensemble average (i.e., expectation value) of the quantity inside the brackets. Likewise, in the expression for $U_{\mathrm{KE}}$ in Equation (2) the quantity ${\dot{x}}^{2}$ occurs, which also needs to be averaged over to obtain $U_{\mathrm{KE}}=\langle U_{\mathrm{KE}}\rangle$. Finally, in the expression for W in Equation (15), $x^{2}$ again appears, as was the case for $U_{\mathrm{PE}}$; this quantity needs to be averaged over to obtain $\langle x^{2}\rangle$ and thereby $W=\langle W\rangle$.

To accomplish these tasks, Ref. [17] first starts with the equation of motion for *q* in Equation (19) and finds the steady state particular solution. This solution contains $E_{\mathrm{rad}}\left(x,\;t\right)$ from Equation (16). $E_{\mathrm{rad}}\left(x,\;t\right)$ provides the stochastic aspects of $x\left(t\right)$ through the Fourier coefficients $A_{p}$, $B_{p}$ and $P_{F}\left(A_{p}\right)=P_{F}\left(B_{p}\right)$ in Equation (20). With that information and the specific form for $E_{\mathrm{rad}}\left(x,\;t\right)$ in Equation (16), $\langle x^{2}\left(t\right)\rangle$ and $\langle {\dot{x}}^{2}\left(t\right)\rangle$ can be found.

As also described in Ref. [17] on pages 236–237, very good approximations can be made using the “continuum approximation” and “a small charge or resonant condition.” $\langle x{\left(t\right)}^{2}\rangle$ then becomes

(21) $$\langle x{\left(t\right)}^{2}\rangle \approx \frac{1}{4\pi m\omega_{0}^{2}}\sigma^{2}\left(\omega_{0},\;T\right)\;\;,$$

where $\sigma^{2}\left(\omega_{0},\;T\right)$ is ${\left(\sigma_{p}\right)}^{2},$ but labelled differently than in Equation (20).

Although not shown explicitly in Ref. [17], the calculation for $\langle {\dot{x}}^{2}\rangle$ proceeds exactly the same as for $\langle x^{2}\rangle$, with the result that

(22) $$\langle \dot{x}{\left(t\right)}^{2}\rangle \approx \frac{1}{4\pi m}\sigma^{2}\left(\omega_{0},\;T\right)\;\;.$$

The other aspect not mentioned in Ref. [17], which is helpful for the present article, is to use

(23) $$\frac{1}{8\pi }\langle E_{\mathrm{rad}}^{2}\left(x,t\right)+B_{\mathrm{rad}}^{2}\left(x,t\right)\rangle =\int_{0}^{\infty }d\omega \rho \left(\omega ,\;T\right)\;\;,$$

to show that

(24) $$\sigma^{2}\left(\omega ,\;T\right)=\frac{\rho \left(\omega ,T\right)c^{3}4\pi^{3}}{\omega^{2}}\;\;.$$

The more familiar quantity in this equation is $\rho \left(\omega ,\;T\right)$, as it is the spectral energy density in units of energy per volume per frequency. It is related, via Equation (23), to $\frac{1}{8\pi }\langle E_{\mathrm{rad}}^{2}\left(x,t\right)+B_{\mathrm{rad}}^{2}\left(x,t\right)\rangle$, which is the electromagnetic energy per unit volume. The less familiar quantity in Equation (24) is $\sigma^{2}\left(\omega ,\;T\right)$, which we have referred to in Equation (20). $\;\sigma^{2}\left(\omega ,\;T\right)$ is the second moment in probabilistic terms of the Fourier coefficients of the radiation. The final results of this study will be expressed in terms of $\rho \left(\omega ,\;T\right)$.

## 4. Heat Flow and Derivation of Classical Electromagnetic ZP Spectrum

Regarding the quantities in Equation (1), let us represent the ensemble average, or expectation value, of the following quantities via

(25) $$U_{\mathrm{KE}}=\langle U_{\mathrm{KE}}\rangle ,\;U_{\mathrm{PE}}=\langle U_{\mathrm{PE}}\rangle ,\;\;W=\langle W\rangle ,\;\;Q=\langle Q\rangle \;\;.$$

From Equations (22), (21), (8) and (24), we have the following:

(26) $$U_{\mathrm{KE}}=\frac{m\langle {\dot{x}}^{2}\rangle }{2}=\frac{1}{8\pi }\sigma^{2}\left(\omega_{0},\;T\right)=\frac{\rho \left(\omega_{0},T\right)c^{3}\pi^{2}}{2\omega_{0}^{2}}\;\;,$$

(27) $$\begin{array}{cc} U_{\mathrm{PE}} & =\frac{q^{2}}{a}+\frac{q^{2}}{4a^{3}}\langle x^{2}\rangle =\frac{q^{2}}{a}+\frac{m\omega_{0}^{2}}{2}\frac{1}{4\pi m\omega_{0}^{2}}\sigma^{2}\left(\omega_{0},T\right)\\ \; & =\frac{q^{2}}{a}+\frac{\rho \left(\omega_{0},T\right)c^{3}\pi^{2}}{2\omega_{0}^{2}}\;\;, \end{array}$$

(28) $$\begin{array}{cc} W & =-\left(\delta a\right)\frac{q^{2}}{a^{2}}\left[1+\frac{3}{4}\frac{\langle x^{2}\rangle }{a^{2}}\right]\\ \; & =-\left(\delta a\right)\left[\frac{q^{2}}{a^{2}}+\frac{3}{4}\frac{q^{2}}{a^{4}}\frac{\sigma^{2}\left(\omega_{0},T\right)}{4\pi m\omega_{0}^{2}}\right]\\ \; & =-\left(\delta a\right)\left[\frac{q^{2}}{a^{2}}+\frac{3}{2a}\frac{\rho \left(\omega_{0},T\right)c^{3}\pi^{2}}{\omega_{0}^{2}}\right]\;\;. \end{array}$$

We can now substitute Equations (26)–(28) into the first law of thermodynamics (Equation (1)):

(29) $$\begin{array}{cc} Q & =\delta \left(U_{\mathrm{KE}}+U_{\mathrm{PE}}\right)-W\\ \; & =\left(\delta a\right)\frac{d}{da}\left[\frac{\rho \left(\omega_{0},T\right)c^{3}\pi^{2}}{2\omega_{0}^{2}}\right]+\left(\delta a\right)\frac{d}{da}\left[\frac{q^{2}}{a}+\frac{\rho \left(\omega_{0},T\right)c^{3}\pi^{2}}{2\omega_{0}^{2}}\right]+\left(\delta a\right)\left[\frac{q^{2}}{a^{2}}+\frac{3}{2a}\frac{\rho \left(\omega_{0},T\right)c^{3}\pi^{2}}{\omega_{0}^{2}}\right]\\ \; & =\left(\delta a\right)c^{3}\pi^{2}\left\{\frac{d}{da}\left[\frac{\rho \left(\omega_{0},T\right)}{\omega_{0}^{2}}\right]+\frac{3}{2a}\frac{\rho \left(\omega_{0},T\right)}{\omega_{0}^{2}}\right\}\;\;. \end{array}$$

Thus, the two terms that do not depend on $\rho \left(\omega_{0},\;T\right)$, namely, $\left(\delta a\right)\frac{d}{da}\frac{q^{2}}{a}$ in the $U_{\mathrm{PE}}$ term and $\left(\delta a\right)\frac{q^{2}}{a^{2}}$ in the *W* term, drop out.

We continue with

(30) $$\begin{array}{cc} Q & =\left(\delta a\right)c^{3}\pi^{2}\left\{\frac{d\omega_{0}}{da}\frac{\partial }{\partial \omega_{0}}\left[\frac{\rho \left(\omega_{0},T\right)}{\omega_{0}^{2}}\right]+\frac{3}{2a}\frac{\rho \left(\omega_{0},T\right)}{\omega_{0}^{2}}\right\}\\ \; & =\;\left(\delta a\right)c^{3}\pi^{2}\left\{\frac{d\omega_{0}}{da}\frac{\partial }{\partial \omega_{0}}\left[\frac{\rho \left(\omega_{0},T\right)}{\omega_{0}^{2}}\right]-\frac{d\omega_{0}}{da}\frac{\rho \left(\omega_{0},T\right)}{\omega_{0}^{3}}\right\}\;, \end{array}$$

since one can show that

(31) $$\frac{d\omega_{0}}{da}=-\frac{3}{2}\frac{\omega_{0}}{a}\;\;.$$

Thus,

(32) $$\begin{array}{cc} Q & =\left(\delta a\right)c^{3}\pi^{2}\frac{d\omega_{0}}{da}\left\{\frac{\frac{\partial }{\partial \omega_{0}}\rho \left(\omega_{0},T\right)}{\omega_{0}^{2}}-\frac{2\rho \left(\omega_{0},T\right)}{\omega_{0}^{3}}-\frac{\rho \left(\omega_{0},T\right)}{\omega_{0}^{3}}\right\}\\ \; & =\left(\delta a\right)\frac{c^{3}\pi^{2}}{\omega_{0}^{3}}\frac{d\omega_{0}}{da}\left\{\omega_{0}\frac{\partial }{\partial \omega_{0}}\rho \left(\omega_{0},T\right)-3\rho \left(\omega_{0},T\right)\right\}\;\;. \end{array}$$

Note that in all these operations, the temperature was held constant such that the change δa was carried out under isothermal conditions.

When Q=0 in Equation (32) such that no heat flows, the solution for Equation (32) is

(33) $$\rho \left(\omega_{0},\;T=0\right)=\kappa \omega_{0}^{3}\;\;.$$

In SED, the constant κ is set equal to $\frac{\hbar }{2\pi^{2}c^{3}}$, where *ℏ* is Planck’s constant. This value of κ enables the predictions of SED to agree with the experimental measurements of the phenomena mentioned at the beginning of this study, such as for Casimir and van der Waals forces.

The reason for switching the argument of ρ from $\left(\omega_{0},T\right)$ to $\left(\omega_{0},T=0\right)$ in Equation (33) is that, for an isothermal operation, there is only one spectrum that can have no heat flow; this is the thermal radiation spectrum at T=0. For this reason, the spectrum of $\rho =\kappa \omega^{3}$ is consequently called the classical electromagnetic zero-point radiation spectrum, as derived here. More specifically, the thermodynamic definition of temperature is (see chapter 7 in Ref. [16] and Ref. [18]) as follows:

(34) $$\frac{Q\;(\mathrm{between}\;S_{1}\;\mathrm{and}\;S_{2}\;\mathrm{at}\;T_{L})}{Q\;(\mathrm{between}\;S_{1}\;\mathrm{and}\;S_{2}\;\mathrm{at}\;T_{H})}=\frac{T_{L}}{T_{H}}\;\;,$$

where $S_{1}$ and $S_{2}$ are adiabatic boundaries, and the heat generation/absorption at temperatures $T_{L}$ and $T_{H}$ are carried out isothermally at those respective temperatures. When $T_{L}=0$, Q(between $S_{1}$ and $S_{2}$ at $T_{L})=0$, as we have in our derivation.

## 5. Entropy and the Second Law of Thermodynamics Applied to the Ring Oscillator

From the second law of thermodynamics, the infinitesimal change in entropy is equal to the following expression, where the heat flow is now properly written as a small, inexact differential carried out over a reversible process (see chapter 7 in Ref. [16]). In contrast, dS is an exact differential:

(35) $$dS=\frac{/\;dQ_{R}}{T}\;\;.$$

From our work thus far, we can write

(36) $$/\;dQ_{R}=dU_{\mathrm{int}}-W\;\;,$$

where we have already calculated $dU_{\mathrm{int}}$ and where

(37) $$W=\delta a\langle F_{\mathrm{avg}\text{-}\mathrm{ext}}\rangle \;\;.$$

$\langle F_{\mathrm{avg}\text{-}\mathrm{ext}}\rangle$ is a rather subtle quantity. It is an ensemble average—hence the $\langle \cdots \rangle$ brackets. However, it is also the average of the force we calculated in Section 2.3, which was a function of θ. We added all angular force contributions due to one single change δa in the radius. Thus, $\langle F_{\mathrm{avg}\text{-}\mathrm{ext}}\rangle$ is the ensemble average of the average of angular force contributions.

In terms of sign, $\langle F_{\mathrm{avg}\text{-}\mathrm{ext}}\rangle$ is always pointed inward to the ring. When δa<0, the external force(s) are doing positive work on our system, and vice versa when δa>0. Hence, the minus sign in Equation (38) is observed below:

(38) $$\langle F_{\mathrm{avg}\text{-}\mathrm{ext}}\rangle =-\left[\frac{q^{2}}{a^{2}}+\frac{3}{2a}\frac{\rho \left(\omega_{0},T\right)c^{3}\pi^{2}}{\omega_{0}^{2}}\right]\;\;.$$

There are two independent thermodynamic coordinates that define the state of our ring-of-charge oscillator system: *a* and T. This contrasts with the much more complicated system in which the analogous equation for the classical electromagnetic ZP radiation spectrum was first derived [14], involving 3N+1 independent thermodynamic coordinates corresponding to *N* electric dipole oscillators (*N* was arbitrary), each having 3 degrees of freedom, plus “1” for temperature [19]. Our present system of the ring-of-charge oscillator is, consequently, far less complicated and likely clearer in terms of the underlying physics.

We can write our infinitesimal change in entropy as

(39) $$dS=\frac{/\;dQ_{R}}{T}=\frac{1}{T}{\left.\frac{\partial U_{\mathrm{int}}}{\partial T}\right|}_{a}dT+\frac{1}{T}\left({\left.\frac{\partial U_{\mathrm{int}}}{\partial a}\right|}_{T}-\langle F_{\mathrm{avg}\text{-}\mathrm{ext}}\rangle \right)da\;\;.$$

From the second law, Equation (39) must be an exact differential. Hence,

(40) $${\left.\frac{\partial S}{\partial T}\right|}_{a}=\frac{1}{T}{\left.\frac{\partial U_{\mathrm{int}}}{\partial T}\right|}_{a}\;\;,$$

(41) $${\left.\frac{\partial S}{\partial a}\right|}_{T}=\frac{1}{T}\left({\left.\frac{\partial U_{\mathrm{int}}}{\partial a}\right|}_{T}-\langle F_{\mathrm{avg}\text{-}\mathrm{ext}}\rangle \right)\;\;.$$

Since dS is an exact differential, we can differentiate Equation (40) with respect to *a* and set it equal to the derivative of Equation (41) with respect to *T*:

(42) $$\begin{array}{cc} \frac{\partial^{2}S}{\partial a\partial T} & =\frac{1}{T}\frac{\partial^{2}U_{\mathrm{int}}}{\partial a\partial T}\\ \; & =\frac{1}{T}\left(\frac{\partial^{2}U_{\mathrm{int}}}{\partial T\partial a}-{\left.\frac{\partial \langle F_{\mathrm{avg}\text{-}\mathrm{ext}}\rangle }{\partial T}\right|}_{a}\right)-\frac{1}{T^{2}}\left({\left.\frac{\partial U_{\mathrm{int}}}{\partial a}\right|}_{T}-\langle F_{\mathrm{avg}\text{-}\mathrm{ext}}\rangle \right)\;\;. \end{array}$$

This results in

(43) $$0=-\frac{1}{T}{\left.\frac{\partial \langle F_{\mathrm{avg}\text{-}\mathrm{ext}}\rangle }{\partial T}\right|}_{a}-\frac{1}{T^{2}}{\left.\frac{\partial U_{\mathrm{int}}}{\partial a}\right|}_{T}+\frac{1}{T^{2}}\langle F_{\mathrm{avg}\text{-}\mathrm{ext}}\rangle \;\;.$$

Note that a term such as ${\left.\frac{\partial \langle F_{\mathrm{avg}\text{-}\mathrm{ext}}\rangle }{\partial T}\right|}_{a}$ above is the same as ${\left.\frac{\partial \langle F_{\mathrm{avg}\text{-}\mathrm{ext}}\rangle }{\partial T}\right|}_{\omega_{0}}$ since $\omega_{0}^{2}=\frac{q^{2}}{2a^{3}m}$; thus, $\omega_{0}$ and *a* are “fixed” with respect to each other.

Thus, it should be mentioned that $U_{\mathrm{int}}$ consists of not only $U_{\mathrm{KE}}+U_{\mathrm{PE}}$ but also the radiation in *V*, in addition to a small coupling between this radiation and our single oscillating charged particle. The notation that signifies these quantities and that spell out their form is $U_{\mathrm{EM},\mathrm{in}}$ and $U_{\mathrm{EM}|\mathrm{in}}$, respectively, in Ref. [14]. However, for a single oscillator, $U_{\mathrm{EM}|\mathrm{in}}$ is a small term that can be ignored. As for $U_{\mathrm{EM},\mathrm{in}}$, it is very weakly affected by changes in *a*, as in ${\left.\frac{\partial U_{\mathrm{int}}}{\partial a}\right|}_{T}$ in Equation (43); this is because $U_{\mathrm{EM},\mathrm{in}}$ mainly contributes via changes in temperature. Consequently, from Equations (26), (27) and (31), we have the following:

(44) $$\begin{array}{cc} {\left.\frac{\partial U_{\mathrm{int}}}{\partial a}\right|}_{T} & ={\left.\frac{\partial }{\partial a}\left(\frac{q^{2}}{a}+\frac{\rho \left(\omega_{0},T\right)c^{3}\pi^{2}}{\omega_{0}^{2}}\right)\right|}_{T}\\ \; & =-\frac{q^{2}}{a^{2}}+c^{3}\pi^{2}\frac{d\omega_{0}}{da}{\left.\frac{\partial }{\partial \omega_{0}}\left[\frac{\rho \left(\omega_{0},T\right)}{\omega_{0}^{2}}\right]\right|}_{T}\\ \; & =-\frac{q^{2}}{a^{2}}-c^{3}\pi^{2}\frac{3}{2}\frac{\omega_{0}}{a}\left[\frac{{\left.\frac{\partial }{\partial \omega_{0}}\rho \left(\omega_{0},T\right)\right|}_{T}}{\omega_{0}^{2}}-2\frac{\rho \left(\omega_{0},T\right)}{\omega_{0}^{3}}\right]\;\;. \end{array}$$

Substituting Equation (44) into Equation (43), plus the expression for ${\left.\frac{\partial \langle F_{\mathrm{avg}\text{-}\mathrm{ext}}\rangle }{\partial T}\right|}_{a}$ and $\langle F_{\mathrm{avg}\text{-}\mathrm{ext}}\rangle$ from Equation (38), results in

(45) $$\begin{array}{cc} 0 & =-\frac{1}{T}\left(-\frac{3}{2a}\frac{{\left.\frac{\partial }{\partial T}\rho \left(\omega_{0},T\right)\right|}_{\omega_{0}}c^{3}\pi^{2}}{\omega_{0}^{2}}\right)-\frac{1}{T^{2}}\left\{-\frac{q^{2}}{a^{2}}-c^{3}\pi^{2}\frac{3}{2}\frac{\omega_{0}}{a}\left[\frac{{\left.\frac{\partial }{\partial \omega_{0}}\rho \left(\omega_{0},T\right)\right|}_{T}}{\omega_{0}^{2}}-2\frac{\rho \left(\omega_{0},T\right)}{\omega_{0}^{3}}\right]\right\}\\ \; & -\frac{1}{T^{2}}\left[\frac{q^{2}}{a^{2}}+\frac{3}{2a}\frac{\rho \left(\omega_{0},T\right)c^{3}\pi^{2}}{\omega_{0}^{2}}\right]\\ \; & =\frac{3}{2}\frac{c^{3}\pi^{2}}{\omega_{0}^{2}}\left\{\frac{1}{aT}{\left.\frac{\partial \rho \left(\omega_{0},T\right)}{\partial T}\right|}_{\omega_{0}}+\frac{1}{aT^{2}}\left[\omega_{0}{\left.\frac{\partial \rho \left(\omega_{0},T\right)}{\partial \omega_{0}}\right|}_{T}-2\rho \left(\omega_{0},T\right)\right]-\frac{1}{T^{2}}\frac{1}{a}\rho \left(\omega_{0},T\right)\right\}\\ \; & =\frac{3}{2}\frac{c^{3}\pi^{2}}{aT^{2}\omega_{0}^{2}}\left[T{\left.\frac{\partial \rho \left(\omega_{0},T\right)}{\partial T}\right|}_{\omega_{0}}+\omega_{0}{\left.\frac{\partial \rho \left(\omega_{0},T\right)}{\partial \omega_{0}}\right|}_{T}-3\rho \left(\omega_{0},T\right)\right]\;\;. \end{array}$$

We observe that the terms drop out that contain $\frac{q^{2}}{a^{2}}$ and that are independent of $\rho \left(\omega_{0},\;T\right)$. This equation must be satisfied by our “ring-of-charge oscillating system” for arbitrary values of *T* and $\omega_{0}$.

The result of Equation (45) is due to the application of the second law of thermodynamics to our ring oscillator system. For an isothermal process, as discussed in Section 4, the term ${\left.\frac{\partial \rho \left(\omega_{0},T\right)}{\partial T}\right|}_{\omega_{0}}$ in Equation (45) equals zero, producing the same result as Equation (32) when *Q* is set equal to zero. For processes that are not isothermal, we have ${\left.\frac{\partial \rho \left(\omega_{0},T\right)}{\partial T}\right|}_{\omega_{0}}\neq 0$, so Equation (45) has a necessary additional term.

Equation (45) is important in the next section of our work on generalizing Wien’s displacement law.

## 6. Derivation of a Generalized Wien’s Displacement Law

In this section, we will stop using the notation of $\omega_{0}$ and instead use ω. Of course, $\omega_{0}$ refers to the resonant frequency of the ring-of-charge oscillator, but by changing *a*, a range of frequencies can be addressed; thus, the simple notation of ω will be used.

The usual form of Wien’s displacement law is that the classical electromagnetic thermal radiation spectrum $\rho \left(\omega ,\;T\right)$ is given by

(46) $$\rho \left(\omega ,\;T\right)=\omega^{3}f\left(\frac{\omega }{T}\right)\;\;.$$

It should be noted that Wien’s law does not specify what the function $f\left(\frac{\omega }{T}\right)$ is but only that *f* must depend on the ratio of $\frac{\omega }{T}$.

One can show that this functional form for $\rho \left(\omega ,\;T\right)$ satisfies Equation (45) from our last section. Specifically,

(47) $$\begin{array}{cc} \; & T{\left.\frac{\partial \rho \left(\omega ,T\right)}{\partial T}\right|}_{\omega }+\omega {\left.\frac{\partial \rho \left(\omega ,T\right)}{\partial \omega }\right|}_{T}-3\rho \left(\omega ,T\right)\\ \; & =T\omega^{3}\frac{\partial f}{\partial \theta }\left(-\frac{\omega }{T^{2}}\right)+\left(\omega 3\omega^{2}f+\omega^{4}\frac{\partial f}{\partial \theta }\frac{1}{T}\right)-3\omega^{3}f=0\;\;. \end{array}$$

However, what is more important about Equation (45) is that it can be used to prove that Equation (46) holds, even when radiation at T=0 exists.

A little background on “Wien’s displacement law” worth mentioning is that most scientists think of it as the law describing how the peak wavelength of blackbody radiation shifts to shorter wavelengths as temperature increases. However, the more primary law is actually Equation (46), from which the peak wavelength scenario can be deduced.

Here, we will derive Equation (46) based on the second law of thermodynamics as applied to our “ring-of-charge oscillator” model. It should be noted, however, that systems other than this ring oscillator model can work equally well, as the key point is to apply the second law to a system with changes in internal energy and with work done on the system.

In contrast to our approach, Wien used a cylinder and a piston, with interior radiation light reflected back and forth between the walls while the piston was pushed into the cylinder’s cavity. Without question, Wien’s method has a strong physical appeal to it, but it is not general enough to include the possibility of ZP radiation or radiation existing at T=0.

In Wien’s and Planck’s work (see the thorough discussion in Ref. [15]), the assumption was made that all radiation vanishes as ω→∞ (see Section 83 in Ref. [15]). This implies, from Equation (46), that radiation vanishes as T→0. We will show that the derivation can be carried out without invoking the demand that thermal radiation vanishes at T→0, which is the point of ZP radiation from Section 4 and the cornerstone of SED. Hence, the generalization of Equation (46) is an important one.

To derive Equation (46) without invoking $\rho \left(\omega ,\;T\right)\to 0$ as T→0, first let $\Theta =\frac{\omega }{T}$ or $T=\frac{\omega }{\Theta }$. Then, we have

(48) $${\left.\frac{\partial \rho \left(\omega ,\frac{\omega }{\Theta }\right)}{\partial \Theta }\right|}_{\omega }={\left.\frac{\partial \rho \left(\omega ,T\right)}{\partial T}\right|}_{\omega }{\left.\frac{\partial T}{\partial \Theta }\right|}_{\omega }\;\;.$$

Solving for ${\left.\frac{\partial \rho \left(\omega ,T\right)}{\partial T}\right|}_{\omega }$ in Equation (48), we have

(49) $$\begin{array}{cc} {\left.\frac{\partial \rho \left(\omega ,T\right)}{\partial T}\right|}_{\omega } & =\frac{1}{{\left.\frac{\partial T}{\partial \Theta }\right|}_{\omega }}{\left.\frac{\partial \rho \left(\omega ,\frac{\omega }{\Theta }\right)}{\partial \Theta }\right|}_{\omega }\\ \; & =-\left(\frac{\Theta^{2}}{\omega }\right){\left.\frac{\partial \rho \left(\omega ,\frac{\omega }{\Theta }\right)}{\partial \Theta }\right|}_{\omega }\;\;. \end{array}$$

Aside from the factor of *T*, this is the first term in Equation (45).

The second term in Equation (45) can be deduced from the first term on the right-hand side (rhs) in the following subtle relationship:

(50) $${\left.\frac{\partial \rho \left(\omega ,\frac{\omega }{\Theta }\right)}{\partial \omega }\right|}_{\Theta }={\left.\frac{\partial \rho \left(\omega ,T\right)}{\partial \omega }\right|}_{T}+{\left.\frac{\partial \rho \left(\omega ,T\right)}{\partial T}\right|}_{\omega }{\left.\frac{\partial \left(\frac{\omega }{\Theta }\right)}{\partial \omega }\right|}_{\Theta }\;\;.$$

Solving for ${\left.\frac{\partial \rho \left(\omega ,T\right)}{\partial \omega }\right|}_{T}$ and substituting Equation (49) into the second term on the rhs in Equation (50) results in

(51) $${\left.\frac{\partial \rho \left(\omega ,T\right)}{\partial \omega }\right|}_{T}={\left.\frac{\partial \rho \left(\omega ,\frac{\omega }{\Theta }\right)}{\partial \omega }\right|}_{\Theta }+\frac{\Theta }{\omega }{\left.\frac{\partial \rho \left(\omega ,\frac{\omega }{\Theta }\right)}{\partial \Theta }\right|}_{\omega }\;\;,$$

which aside from a factor of ω gives the second term in Equation (45).

Substituting Equations (49) and (51) into Equation (45) then yields

(52) $$\begin{array}{cc} 0 & =\left(\frac{\omega }{\Theta }\right)\left(-\frac{\Theta^{2}}{\omega }\right){\left.\frac{\partial \rho }{\partial \Theta }\right|}_{\omega }+\omega \left({\left.\frac{\partial \rho }{\partial \omega }\right|}_{\Theta }+\frac{\Theta }{\omega }{\left.\frac{\partial \rho }{\partial \Theta }\right|}_{\omega }\right)-3\rho \\ \; & =\omega {\left.\frac{\partial \rho }{\partial \omega }\right|}_{\Theta }-3\rho \;\;. \end{array}$$

This result looks very similar to Equation (32) with Q=0. However, there is an important difference, and that is the term ${\left.\frac{\partial \rho }{\partial \omega }\right|}_{\Theta }$, with the variable Θ held fixed during differentiation with respect to ω. For a fixed ratio of $\frac{\omega }{T}$, $\rho \left(\omega ,\;T\right)$ must depend upon ω by being proportional to $\omega^{3}$. Thus, in general, we can conclude that

$$\rho \left(\omega ,\;T\right)=\omega^{3}f\left(\frac{\omega }{T}\right)\;\;,$$

with the function *f* being an unspecified function; other than this, it depends on $\frac{\omega }{T}$. This result, obtained using the second law of thermodynamics, is the same as Wien’s displacement law, but its derivation does not make the implicit assumption that ρ→0 as T→0.

To illustrate Equation (46) being adhered to in “candidates” for thermal radiation spectra, here are three such examples. The first example is the Rayleigh–Jeans spectrum:

(53) $$\rho_{\mathrm{RJ}}=\frac{\omega^{2}}{c^{3}}\left(\frac{k_{B}T}{\pi^{2}}\right)=\omega^{3}\left(\frac{k_{B}}{c^{3}\pi^{2}\left(\frac{\omega }{T}\right)}\right)\;\;,$$

where $k_{B}$ is Boltzmann’s constant.

The second is the ZP radiation spectrum, which is valid at T=0:

(54) $$\rho_{\mathrm{ZP}}=\frac{\omega^{2}}{c^{3}}\left(\frac{\hbar \omega }{2\pi^{2}}\right)=\omega^{3}\left(\frac{\hbar }{c^{3}2\pi^{2}}\right)\;\;.$$

The third is the ZP plus Planckian spectrum (ZPP):

(55) $$\rho_{\mathrm{ZPP}}=\frac{\omega^{2}}{c^{3}}\left[\frac{\hbar \omega }{2\pi^{2}}\coth\left(\frac{\hbar \omega }{2k_{B}T}\right)\right]=\omega^{3}\left\{\frac{\hbar }{c^{3}2\pi^{2}}\coth\left[\frac{\hbar }{2k_{B}}\left(\frac{\omega }{T}\right)\right]\right\}\;\;.$$

Another equivalent method of writing $\rho_{\mathrm{ZPP}}$, which is likely to be more familiar to people, is

(56) $$\begin{array}{cc} \rho_{\mathrm{ZPP}} & =\frac{\omega^{2}}{c^{3}}\left\{\frac{1}{\pi^{2}}\left[\frac{\hbar \omega }{2}+\frac{\hbar \omega }{\exp\left(\frac{\hbar \omega }{k_{B}T}\right)-1}\right]\right\}\\ \; & =\omega^{3}\left(\frac{\hbar }{c^{3}2\pi^{2}}\left\{1+\frac{2}{\exp\left[\frac{\hbar }{k_{B}}\left(\frac{\hbar }{T}\right)\right]-1}\right\}\right)\;\;. \end{array}$$

As can be seen, each of these spectra can be written in the form of Equation (46). It should be noted that $\lim_{T\to 0}\rho_{\mathrm{ZPP}}\left(\omega ,\;T\right)=\rho_{\mathrm{ZP}}\left(\omega \right)$. Moreover, the reason for writing $\frac{\omega^{2}}{c^{3}}$ as a factor in the first term of each line is that $\frac{\omega^{2}}{c^{3}}$ is related to the number of normal modes.

The reason why $\rho_{\mathrm{ZP}}$ is important in SED is that it enables correct predictions for a large range of phenomena, as mentioned at the beginning of this study. However, $\rho_{\mathrm{ZPP}}$ is also quite important, such as for showing agreement at all temperatures for SHO behavior compared with QM and QED [8]. Moreover, the van der Waals forces at all distances and temperature conditions have been carried out [7]. Casimir forces as a function of temperature are treated in Ref. [6]. Another example of the use of $\rho_{\mathrm{ZPP}}$ is in the thermodynamics of radiation in a cavity [10].

As for $\rho_{\mathrm{RJ}}$, this was a spectrum deduced by Rayleigh and Jeans around 1900, during which time Planck carried out blackbody radiation research. It is accurate for long wavelengths for radiation in a black body but fails substantially for shorter wavelengths. Thus, RJ radiation presents many problems, one of the most serious being that it predicts an infinite specific heat [19]. Specifically, one cannot make even an infinitesimal change in temperature without an infinite energy change required. Moreover, RJ radiation violates the third law of thermodynamics [14].

## 7. Thermodynamic Temperature Ratio

The definition of the temperature ratio in Equation (34) is clearly an important point, particularly for understanding the T=0 case. The unusual situation for T=0 is that it is both an isotherm (*T* is constant during the thermodynamic process) and an adiabatic curve (no heat flows during the thermodynamic process). If T>0, then the use of Equation (32), without setting it equal to zero, is the correct equation for obtaining the heat generated in each of the two isotherms in Equation (34). However the endpoints of each isotherm is an adiabatic surface, with one side of each isotherm ending in the same adiabatic surface and the other side of each isotherm ending in another, but same for each, adiabatic surface.

These adiabatic curves can be obtained by first setting (32) to equal zero; thus, the adiabatic condition is realized, and no heat is emitted or absorbed in *V*. Secondly, the term ${\left.\frac{\partial \rho \left(\omega_{0},T\right)}{\partial \omega_{0}}\right|}_{T}$ should be changed to

$${\left.\frac{\partial \rho \left(\omega_{0},T\right)}{\partial \omega_{0}}\right|}_{T}+\frac{dT}{d\omega_{0}}{\left.\frac{\partial \rho \left(\omega_{0},T\right)}{\partial T}\right|}_{\omega_{0}}\;\;,$$

since changes in $\omega_{0}$, meaning *a* as well, now affect the temperature. In other words, the following equation will generate the adiabatic curves needed to border the isotherms:

(57) $$0=-3\rho \left(\omega_{0},\;T\right)+\omega_{0}\left[{\left.\frac{\partial \rho \left(\omega_{0},T\right)}{\partial \omega_{0}}\right|}_{T}+\frac{dT}{d\omega_{0}}{\left.\frac{\partial \rho \left(\omega_{0},T\right)}{\partial T}\right|}_{\omega_{0}}\right]\;\;.$$

One can then solve for $\frac{dT}{d\omega_{0}}$ and use that to generate the adiabatic curves.

In addition to the above, some criteria need to be imposed such that the radiation used in Equation (34) is of a thermal character. Although the Wien displacement law does not fully identify the proper thermal spectrum (an infinite number of candidates are still allowed due to the nonspecific function $f\left(\frac{\omega }{T}\right)\;$), one still needs to impose it to obtain the proper ratio in Equation (34).

Reference [18] explains the specifics of carrying out these calculations in more detail, in addition to how the ZP situation fits within this analysis and its relation to the Carnot processes.

## 8. Concluding Remarks

An unusual oscillating system is described here, which consists of a ring of charge with a single charged point particle near the center. The charged particle has the same charge sign as the ring of charge. Consequently, as the particle approaches one part of the ring, the increased electrostatic force pushes the particle back toward the center of the ring. The result is an oscillatory motion that exhibits SHO behavior for small displacements. In addition, however, we assumed that classical electromagnetic thermal radiation was present. Electromagnetic radiation exerts a fluctuating force on the charged particle that is modified by the resonant effect of the SHO potential from the ring of charge.

Calculating the heat passing into or out of a large volume that encloses this oscillating system during a thermodynamic isothermal and reversible operation enables a criterion to be set and examined, namely, when the heat equals zero. This is the property needed for the system to be at absolute zero temperature, which allows us to derive the classical electromagnetic ZP radiation spectrum. Since ZP radiation is a foundation for SED, being able to derive this spectrum is clearly important for SED.

The second law of thermodynamics was applied regarding the entropy of this system in Section 5 and Section 6. This resulted in a “generalized Wien’s displacement law” in Section 6. More specifically, the usual functional form for the thermal radiation spectrum of $\omega^{3}f\left(\frac{\omega }{T}\right)$ was deduced around 1990, with the explicit assumption that thermal radiation vanishes as T→0. Obviously, this does not fit within the main context of this article. A new derivation for Wien’s displacement law was carried out, which did not make the assumption that thermal radiation vanishes at T=0. Not making this assumption, but still deducing the functional form of $\omega^{3}f\left(\frac{\omega }{T}\right)$, means that ZP radiation, as well as ZPP radiation, fits within the Wien law criteria for thermal radiation.

Finally, an outline was provided on how to include this information within a Carnot-like set of processes for the thermal temperature ratio of Equation (34), in addition to methods for the adiabatic surfaces needed in this heat/temperature ratio.

The electrodynamic oscillating model in this study greatly simplified the first derivation [14] for the ZP spectrum based on no heat flow during displacement operations. The derivation in Ref. [14] involved the slow displacement of one or more of *N* fluctuating electric dipoles. The interaction between the electric dipoles was that of van der Waals forces, valid for all distances. Consequently, the analysis was considerably more complicated than that in the present study, enabling the present study, in many ways, to offer clearer physical insight. Another important difference between the research conducted here and the earlier research in Refs. [10,14] is that the present study involved work done directly on displacing the structure that modifies the oscillator’s resonant frequency. Here, the structure was the radius of the ring of charge. Other studies have involved much more indirect resonant frequency effects.

The first major property of ZP radiation was discovered in the 1960s: ZP radiation is Lorentz-invariant [20,21]. It is interesting that the property discussed in the present study was found much later, in the early 1990s: namely, no heat flow during isothermal, reversible thermodynamic operations in ZP radiation [10,14].

## Data Availability

All data regarding this article is contained in the article itself.
